# ACE2 deficiency inhibits thoracic aortic dissection by enhancing SIRT3 mediated inhibition of inflammation and VSCMs phenotypic switch

**DOI:** 10.1186/s10020-024-00926-4

**Published:** 2024-09-19

**Authors:** Liqing Jiang, Linhe Lu, Chao Xue, He Sun, Kai Ren, Liyun Zhang, Hanzhao Zhu, Bin Zhang, Xiaoya Wang, Xinan Qiao, Xiangyan Peng, Jincheng Liu, Weixun Duan

**Affiliations:** Department of Cardiovascular Surgery, Xijing Hospital, Air Force Military Medical University, 127 Changle West Road, Xi’an, 710032 People’s Republic of China

**Keywords:** Thoracic aortic dissection, ACE2, SIRT3, Vascular smooth muscle cell, Phenotypic switch, Inflammation

## Abstract

**Background:**

Thoracic aortic dissection (TAD) is an irreversible cardiovascular disorder with high mortality and morbidity. However, the molecular mechanisms remain elusive. Thus, identifying an effective therapeutic target to prevent TAD is especially critical. The purpose of this study is to elucidate the potential mechanism of inflammation and vascular smooth muscle cell (VSMCs) phenotypic switch in β-aminopropionitrile fumarate (BAPN)-induced TAD.

**Methods:**

A mouse model of TAD induced by BAPN and IL-1β -stimulated HVSMCs in vivo and in vitro models, respectively. ACE2 Knockdown mice treated with BAPN or without, and the TAD mouse model was treated with or without AAV-ACE2. Transthoracic ultrasound was conducted for assessment the maximum internal diameter of the thoracic aorta arch. RNA sequencing analysis was performed to recapitulate transcriptome profile changes. Western blot were used to detect the expression of MMP2, MMP9, ACE2, SIRT3, OPN, SM22α and other inflammatory markers. The circulating levels of ACE2 was measured by ELISA assay. Histological changes of thoracic aorta tissues were assessed by H&E, EVG and IHC analysis.

**Results:**

We found that circulating levels of and the protein levels of ACE2 were increased in the TAD mouse model and in patients with TAD. For further evidence, ACE2 deficiency decelerated the formation of TAD. However, overexpression of ACE2 aggravated BAPN-induced aortic injury and VSMCs phenotypic switch via lowered SIRT3 expression and elevated inflammatory cytokine expression.

**Conclusion:**

ACE2 deficiency prevented the development of TAD by inhibiting inflammation and VSMCs phenotypic switch in a SIRT3-dependent manner, suggesting that the ACE2/SIRT3 signaling pathway played a pivotal role in the pathological process of TAD and might be a potential therapeutical target.

**Supplementary Information:**

The online version contains supplementary material available at 10.1186/s10020-024-00926-4.

## Highlights

1. This study demonstrated for the first time that ACE2 deficiency attenuates the development of TAD induced by BAPN.
2. The inhibitory effect of ACE2 deficiency on phenotypic transformation of VSMCs and inflammation may be through SIRT3 signaling pathway.
3. Specific inhibition of SIRT3 can speed the exacerbation of TAD induced by BAPN and SIRT3 may be an important target for drug therapy of TAD.

**Supplementary Information:**

The online version contains supplementary material available at 10.1186/s10020-024-00926-4.

## Introduction

TAD is an irreversible cardiovascular disorder involving all three layers of the vascular wall. Character by artery dilation, TAD carries a high risk of mortality and morbidity, even with advanced treatment (Xia et al. 2020; Luo et al. 2020; Sakalihasan et al. 2018). The main features of TAD include ECM degradation, inflammation, and loss of MSCs and phenotypic transformation by tearing of the inner layer (Luo et al. 2020; Zhang et al. 2022b, a). However, no effective clinical medications have been proven to prevent TAD (Chen et al. 2022). Thus, identifying an effective therapeutic target to prevent TAD is especially critical, although the molecular mechanisms of TAD remain elusive.

As an important component of the renin-angiotensin system (RAS) system, angiotensin converting enzyme 2 (ACE2) plays a pivotal role in the homeostatic control of cardiovascular function, and a growing body of evidence highlights the importance of the ACE2 family in the pathogenesis of arterial diseases (Drucker 2020; Patel et al. 2016). Previous studies have confirmed that the expression of ACE2 is decreased in VSMCs in hypertrophic cardiomyopathy and dilated cardiomyopathy (Chung et al. 2021). ACE2 deficiency has been shown to exacerbate angiotensin II-induced vascular remodeling through increased VSMC loss (Patel et al. 2014; Li et al. 2015). In addition to its well-known function as an angiotensin-converting enzyme, the protease-independent functions of ACE2 have also been increasingly recognized. Unlike the pathogenesis of aortic aneurysm and dissection induced by AngII, the pathogenesis of TAD induced by BAPN involves the inhibition of lysyl oxidase and lysyl oxidase-like proteins, which are required for elastin and collagen crosslinking, a critical process in ECM development and maturation (Sawada et al. 2022). However, the involvement and functional roles of ACE2 in the setting of TAD induced by BAPN remain largely unknown.

Sirtuin3 (SIRT3) is a histone deacetylase involved in the pathological process of various cardiovascular diseases (Zhang et al. 2020). Dikalova demonstrated that SIRT3 deficiency increased vascular dysfunction by enhancing inflammation and oxidative stress in hypertension (Dikalova et al. 2020). Importantly, SIRT3 plays a pivotal role in reducing vascular inflammation, reactive oxygen species, and apoptosis in VSMCs against TAD (Qiu et al. 2021). As reported in the literature, ACE2 and sirtuin 1 have been implicated in the formation of aortic aneurysms (Moran et al. 2017). Here, we expanded on these findings to clarify the role of ACE2 deficiency in the pathological process and its relationship with SIRT3. A growing body of evidence has showed that pro-inflammatory cytokine upregulation is observed in mouse and human TAD specimens, with increased inflammatory cell infiltration into the aortic adventitia was increased and playing a critical role in vascular inflammation and destruction (Luo et al. 2020; Zhang et al. 2022b, a; Piao et al. 2021). Furthermore, the NLRP3 (the NOD-, LRR-, and pyrin domain-containing protein 3) inflammasome is one of the best characterized inflammasomes, involved in SMC contractile dysfunction in TAD, which must be tightly regulated to avoid excessive inflammation (Wu, et al. 2017; Hooftman, et al. 2020). Despite advances in our understanding of the interactions between NLRP3 and TAD, a unifying molecular pathway of the upstream of NLRP3 remains to be elucidated. It is reported that activating SIRT3 can subsequently inhibit the NLRP3 inflammasome cascade to attenuate myocardial ischemia–reperfusion injury (Zhang et al. 2022b, a).

The present study aims to determine the relationship between the ACE2/SIRT3 signaling pathway and NLRP3-mediated inflammation in TAD.

## Materials and methods

### Clinical specimens

This study was approved by the Xijing Hospital of Air Force Military Medical University. Peripheral blood was collected from patients with TAD (n = 20) or hypertension (n = 20) from the Xijing Hospital. The human aortic tissue was collected from patients with TAD and heart transplant donors. The heart donors were age-matched patients undergoing heart transplant surgery without aortic aneurysm, dissection, coarctation, or previous aortic repair. The human tissue samples were performed accordance with the relevant guidelines and regulations. The protocol for human specimens were approved by the ethics committee of Xijing Hospital. Written informed consent forms were obtained from the patients or the donors’ families.

### Animals and ethics statement

C57BL/6J mice (male, 3-week-old) were obtained from the Laboratory Animal Center of Air Force Medical University and maintained in a temperature-controlled barrier facility under 12:12 h dark-and-light cycles. Ace^−/−^ mice (S-KO-13234) were obtained from Cyagen Biosciences (Guangzhou, China) and were backcrossed onto a C57BL/6J (Pan et al. 2018). The generations of knockout mice were confirmed by polymerase chain reaction (PCR) using genotyping primers presented in supplementary materials. The animal protocols were confirmed to the 2019 Guide for the Care and Use of Laboratory Animals.

### Reagents

BAPN was purchased from Sigma Aldrich (A3134, St. Louis, MO, USA), Elastic Verhoeff-van-Giesen (EVG) staining kit was obtained from Leagene Biotech (Beijing, China), 3-TYP (HY-108331) and Compound 5v (HY-158426) were obtained from MedChemexpress Biotechnology ( Shanghai, China), α-SMA (ab5694), ACE2 (ab108252), MMP9 (ab283575), MMP2 (ab92536), NLRP3 (ab214185), IL-1β (ab254360), OPN (ab283656), SM22α (ab14106), SOD2 (ab13141s), Ac-SOD2 (ab137037) antibody were purchased from Abcam (Cambridge, MA, USA), SIRT3 (10099-1-AP), TNF-α (17590-1-AP), GAPDH (10494-1-AP) antibody were purchased from PEPROTECH (Rocky Hill, NJ, USA), antibody against CD68 (sc-9139) were purchased from Santa Cruz Biotechnology (Dallas, TX, USA), and 1L-6 (12912) antibody was purchased from Cell Signaling Technology (Danvers,USA). Human ACE2 (A5KN6N5T4R), Mouse AngII (E-EL-M2612) ELISA kits were purchased from Elabscience (Wuhan, China). Human recombinant IL-1β (0606B95 L0821) was purchased from PEPROTECH (Rocky Hill, NJ, USA).

### In vivo experiments

Three-week-old mice were randomly divided into the following groups, and a TAD mouse model was established as previously described (Pan et al. 2021). Briefly, three-week-old C57/BL mice were fed with normal chow diet and administered freshly prepared BAPN dissolved in drinking water at a dose of 1 g/kg/day for 4 weeks. Subsequently, the mice were randomly divided into different groups according to experimental requirements (Liu et al. 2017). Mice were injected via the tail vein with AAV (adeno-associated virus) ACE2 or AAV null, and fed with normal drinking water or treated with BAPN. 3-TYP, the SIRT3 inhibitors was used to intraperitoneal injection into mice (50 mg/kg/time, once/2 days).

### Adeno-associated virus infection

Recombinant AAV9 with SM22a promotor carrying ACE2 or ACE2 adenovirus (Ad) was constructed by Hanbio Biotechnology Co., Ltd. The sequence of ACE2 RNA was CDS region sequence of NM_027286 transcript. 100 μL of AAV (1.3 × 10^12^ drips/mL) was administered via tail intravenous injection for 3 week-old mice. Negative control (AAV-Null) was injected as a control.

### Serum cytokines detection

Human serum ACE2 levels were measured by ELISA kits according to the manufacturer’s instructions. Finally, absorbance values were determined using a microplate reader (SpectraMax M5 plate reader, Molecular Devices, Sunnyvale, California, USA).

### Ultrasonography monitoring

Transthoracic ultrasound (2-dimensional) was conducted for aorta morphology assessment using a Vevo 2100 imaging system (VisualSonics, Toronto, Canada) equipped with a 30-MHz linear transducer. The maximum internal diameter of the thoracic aorta was measured with longitudinal images of aortic arch. It is worth noting that mice were anesthetized by inhalation of 1.5–2% isoflurane with the maintenance of stable body temperature at the ultrasound station as we previously described (Xia et al. 2020).

### Blood pressure measurement

Systolic blood pressure of mice were measured using a non-invasive small animal blood pressure monitoring system (CODA Monitor, Torrington, America). Briefly, the system parameters and corresponding experimental series were set on the detection software interface. Then mice were placed in the holder in a quiet room and warmed with a constant temperature electric blanket. The mice tail root was positioned in the sensor of the blood pressure meter and should fit closely. Five measurements were recorded from each mouse, and the average of 5 measurements was reported as the pressure value of each mouse.

### Histological analysis

At the end of the experiment, the histological morphology of the entire aorta was observed by hematoxylin and eosin (H&E) and elastic Van Gieson (EVG) staining after the tissue was fixed in 4% paraformaldehyde, embedded in paraffin, and sectioned into 4–5 μm thick slices. Briefly, sections were soaked in hematoxylin staining solution for 5 min, rinsed with distilled water, and then soaked in hydrochloric ethanol for a few seconds. They were stained with eosin staining solution for 30 s, and finally rinsed with distilled water for 5 min. and the sections were then sealed with neutral resin after dehydration and clearing were conducted. EVG staining was used for scoring the degradation of the medial elastic lamina according to the elastin degradation-grading (no, mild, and severe degradation, even aortic rupture). In brief, the sections were soaked in hematoxylin solution and washed with distilled water after dewaxing and hydration. Subsequently, the sections were deiodination and re-stained with Van Gieson solution. The next operation steps are the same as H&E staining. All images were obtained using a microscope (Olympus, Japan).

### Immunohistochemistry and immunofluorescence assay

α-SMA staining score was used for evaluating the severity of the loss of VSMC and ACE2 double-staining was conducted for the localization of ACE2. Then, Next, 4',6-diamidino-2-phenylindole (DAPI) was used to counterstain the nucleus. Images of the sections were acquired immediately and examined using an Olympus Fluoview FV1000 microscope (Olympus, Japan). The sections were incubated with 3% hydrogen peroxide and 10% goat serum for 30 min, and CD68, OPN, SM22α, SIRT3, and α-SMA staining were performed, and all images were obtained using a microscope (Olympus, Japan). The Image J pro plus software was used for the quantification the number of positive cells.

### RNA sequencing

Thoracic aorta tissue from mice in the control group and BAPN group were used for RNA isolation, library construction, and RNA sequencing. Total RNA was isolated with Trizol reagent according to the manufacturer’s protocol. DESeq2 software for this experiment was used to screen for differentially expressed genes (DEGs) between different sample groups, and DEGs with | log2FC | at least > 1 and P-value < 0.05 were considered to be significant.

### In vitro experiments

Human primary VSMCs were obtained from thoracic aortic tissues from TAD patients and donors. The tissue was removed and washed 3 times with pre-cooled PBS, transferred in DMEM medium and cut into 1 mm^3^ pieces. It was then treated with 1 mg/mL collagenase I (EZ4567C110, Sigma Aldrich, St. Louis, Missouri, USA) for 3 h. Next, the tissue suspension was transferred into a culture flask with a pipette, and the collagenase I liquid was aspirated. The tissue pieces are spread flat in the culture flask. Flip the adherent flask and maintain for 3 h in culture incubator. After the tissue pieces is completely attached to the wall, flip the culture flask and add the DMEM containing 20% FBS for 3–4 weeks. Positive cells of α-SMA immunofluorescence were considered successful extraction. VSMCs were infected with ACE2 or NC adenovirus for up to 24 h. Afterwards, the cells were treated with/without recombinant human IL-1β 10 ng/mL (Alesutan et al. 2021), and simultaneously added SIRT3 activator (Compound 5v) for 24 h.

### Western blot

The total protein of human or murine aortic tissues were prepared by using a RIPA lysis buffer and stored at − 80 ℃. Equal amount of protein (40 μg protein) was separated by using the sodium dodecyl sulfate–polyacrylamide gel electrophoresis and then the proteins were transferred to a polyvinylidene difluoride membrane (Merck Millipore, Darmstadt, Germany). Afterwards, the membranes were incubated with 5% non-fat milk for 2 h at room temperature and then incubated with primary and secondary antibodies. Finally, the bands were visualized by a ChemiDoc system (Bio-Rad, Richmond, CA, USA) and the signal was quantified by Image Lab software (Bio-Rad, Richmond, CA, USA). It should be noted that β-actin or glyceraldehyde-3-phosphate dehydrogenase (GAPDH) were considered as the internal loading control according to the molecular weight of target proteins. Each experiment was repeated three times at least.

### Statistical analysis

All data were presented as mean ± SEM and were analyzed with the GraphPad Prism Software version 9.0 (GraphPad Software, San Diego, CA, USA). The differences between the two groups were compared by using a two-tail t-test. And the differences among three or more groups were compared by using one- or two-way ANOVA and Bonferroni correction. The differences in murine mortality and TAD accidence or baseline data between patients with TAD or the normal control group were compared by using the chi-square test. And P < 0.05 was considered statistically significant.

## Results

### ACE2 is up-regulated in the pathological process of TAD

**Fig. 1:**
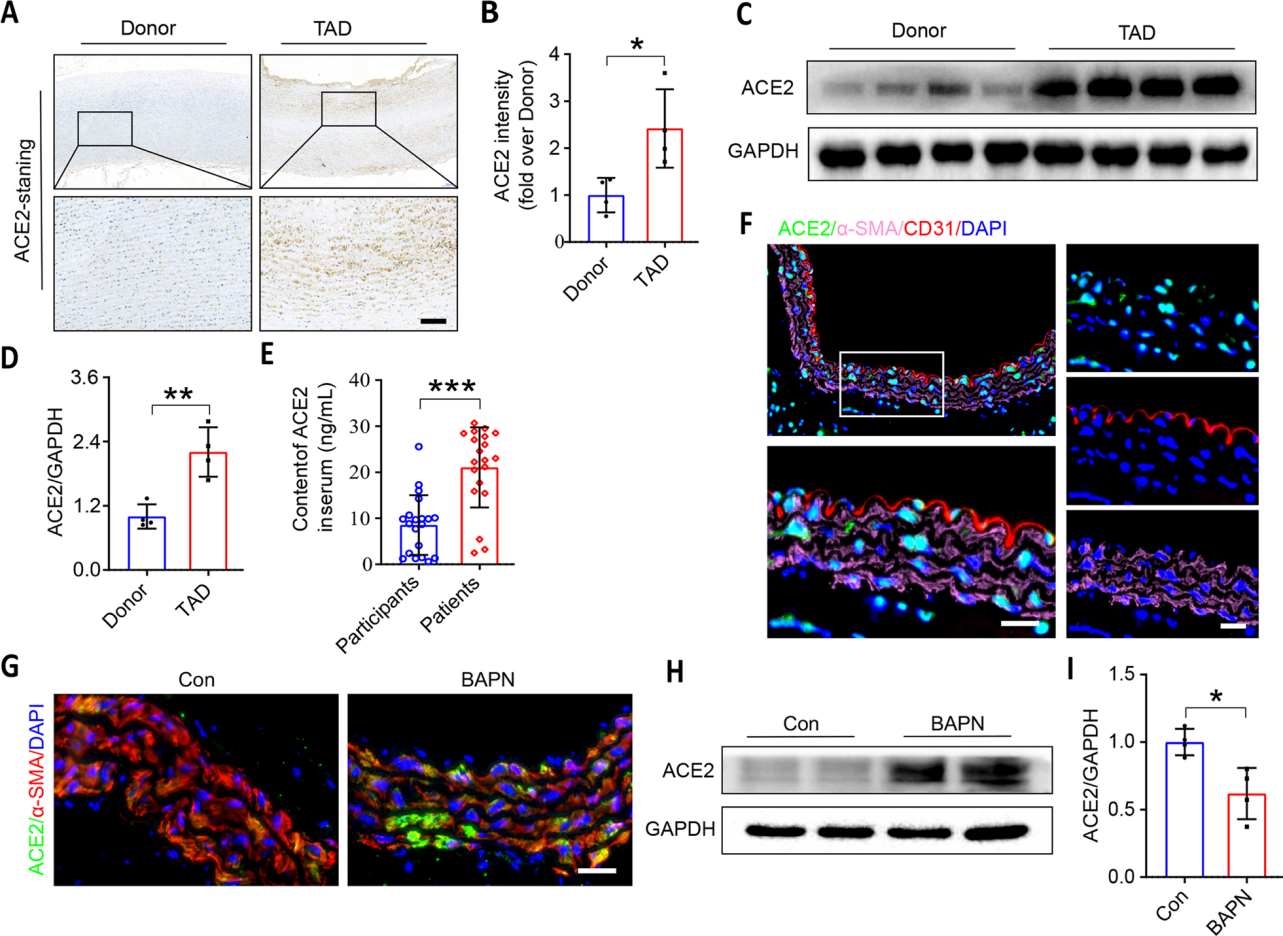
Staining of main pathological changes in aortic sections, ACE2 is activated in TAD patient and BAPN induced mice

To evaluate the association between ACE2 and TAD, aortic pathological specimen examination was conducted in patients with TAD and heart transplant donor. Peripheral blood plasma was tested in patients with TAD and participants diagnosed with hypertension but no other diseases, the clinical data was listed in Table 1. As shown in Fig. 1A–E, ACE2 immunohistochemistry staining, peripheral blood plasma detection and western blotting demonstrated that the expression of ACE2 was upregulated in patients with TAD than donor or participants. Furthermore, immunofluorescence staining showed that ACE2 was mainly localized in VSMC (Fig. 1F). In addition, ACE2 was also up-regulated in the aortic walls of BAPN-induced mice, as confirmed by immunofluorescence staining and Western blotting (Fig. 1G–I).

**Table 1:** Baseline demoraphics of Patients for plasma sources

Characteristics	Hypertensive patients (n = 20)	TAD patients (n = 20)
Age	50.00 ± 8.28	48.60 ± 11.64
Man/Female (n)	15/5	16/4
Drinking history, n (%)	4, (20%)	3, (15%)
Smoking history, n (%)	11, (55%)	6, (30%)
Medical history, n( %)	0, (0%)	0, (0%)
Hypertensive, n (%)	20, (100%)	10, (50%)
Diabetes, n (%)	0, (0%)	0, (0%)
Hyperlipidemia, n (%)	0, (0%)	0, (0%)

As previously reported, TAD dramatically impaired the histological morphology of thoracic aorta compared with the donor group (Fig. S2A). Moreover, EVG staining depicted that the degradation of elastic fibres was exacerbated in patients suffering from TAD, with the significantly increased of elastin scores (Fig. S2B and 2D). These alterations may be partially caused by upregulation and activation of the ECM metalloproteinases such as matrix metalloproteinases 2 and 9(Fig. S2F-H). In addition, compared with the donor group, the number of CD68-positive cells was substantially increased in the thoracic aorta specimens (Fig. S2C and 2E). Collectively, these findings suggest that ACE2 is increased, degradation of ECM, and infiltration of inflammatory cells contribute to TAD formation.

### VSMCs phenotypic modulation is the main pathological feature of TAD and inflammation promotes phenotype transformation of VSMCs

**Fig. 2:**
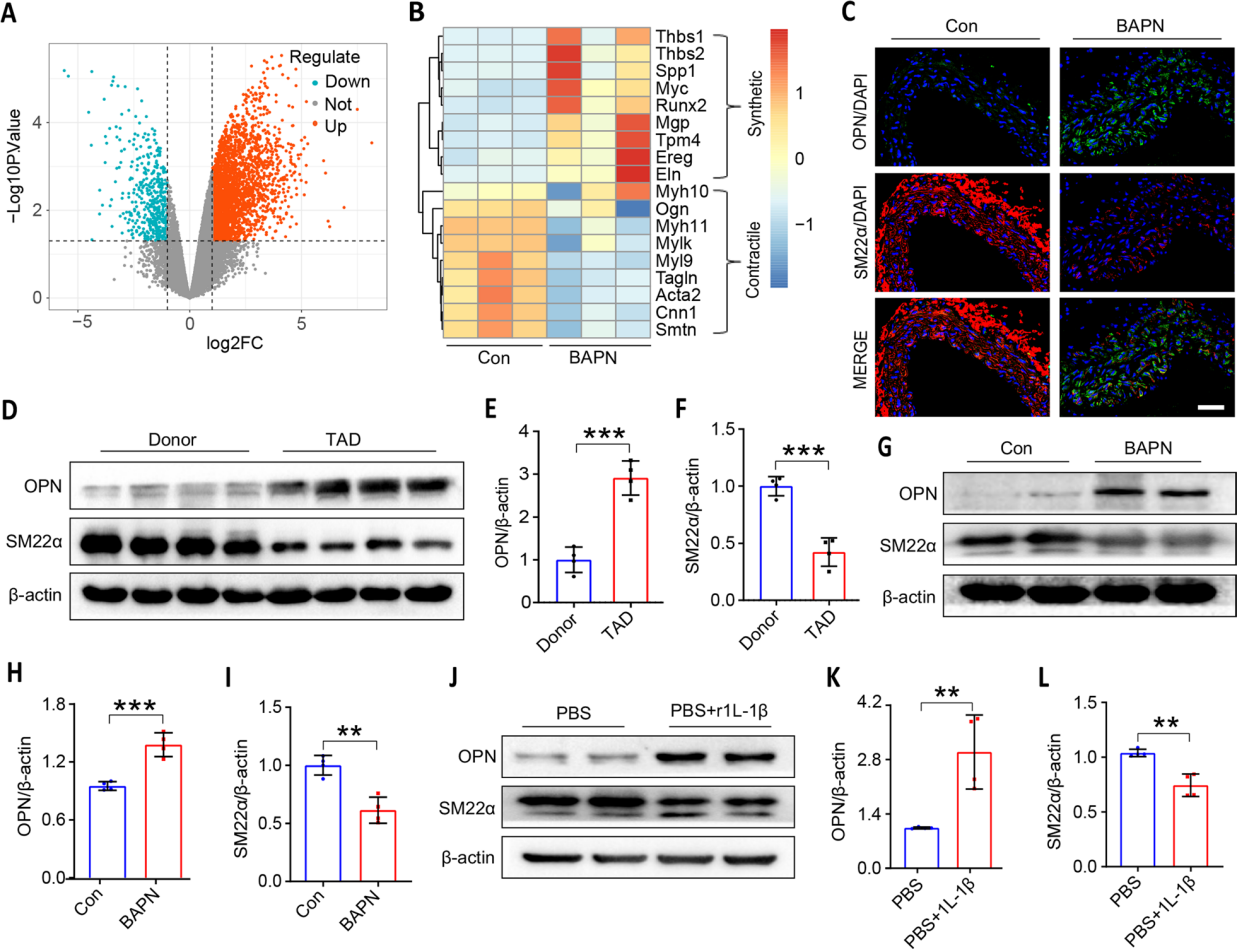
VSMCs undergo phenotypical switching in TAD patient and BAPN-induced mice, and human VSMCs exposed to 1L-1β undergo phenotypic switching

As is well known, VSMCs switching from a contractile phenotype to a synthetic phenotype promote a pro-inflammatory response and increased MMPs production, which results in TAD progression. A murine TAD model was established using 4-week BAPN administered orally in C57BL/6J mice. Thus, a volcano plot showed numerous differentially expressed transcripts between the control group and the BAPN group (Fig. 2A). Among all these differentially expressed genes, the hierarchical clustering heatmap identified differentially expressed genes associated with SMC contractile (*Tagln, Acta2, Cnn1, Smtn*) and synthetic (*spp1, Tmp4, Ereg, Eln*) phenotypes (Fig. 2B). Meanwhile, immunofluorescence staining in Fig. 2C showed that the VSMC synthetic marker OPN was increased and contractile marker SM22α was down-regulated in thoracic aortic tissues of mice in the BAPN group compared with the control group. Similarly, western blot results from clinical and in vivo mouse specimens demonstrated that the changes in SM22α and OPN had the same trend with the immunofluorescence data (Fig. 2D–I). Interestingly, we treated human VSMCs with IL-1β and also found that the contractile marker SM22α was reduced and the synthetic marker OPN was increased (Fig. 2J–L). Those results confirmed that VSMCs switching from contractile phenotype to synthetic phenotype is the main pathological feature of TAD, and inflammation promotes the VSMCs phenotypic switch.

### Knock out of ACE2 attenuates the development of TAD induced by BAPN

**Fig. 3:**
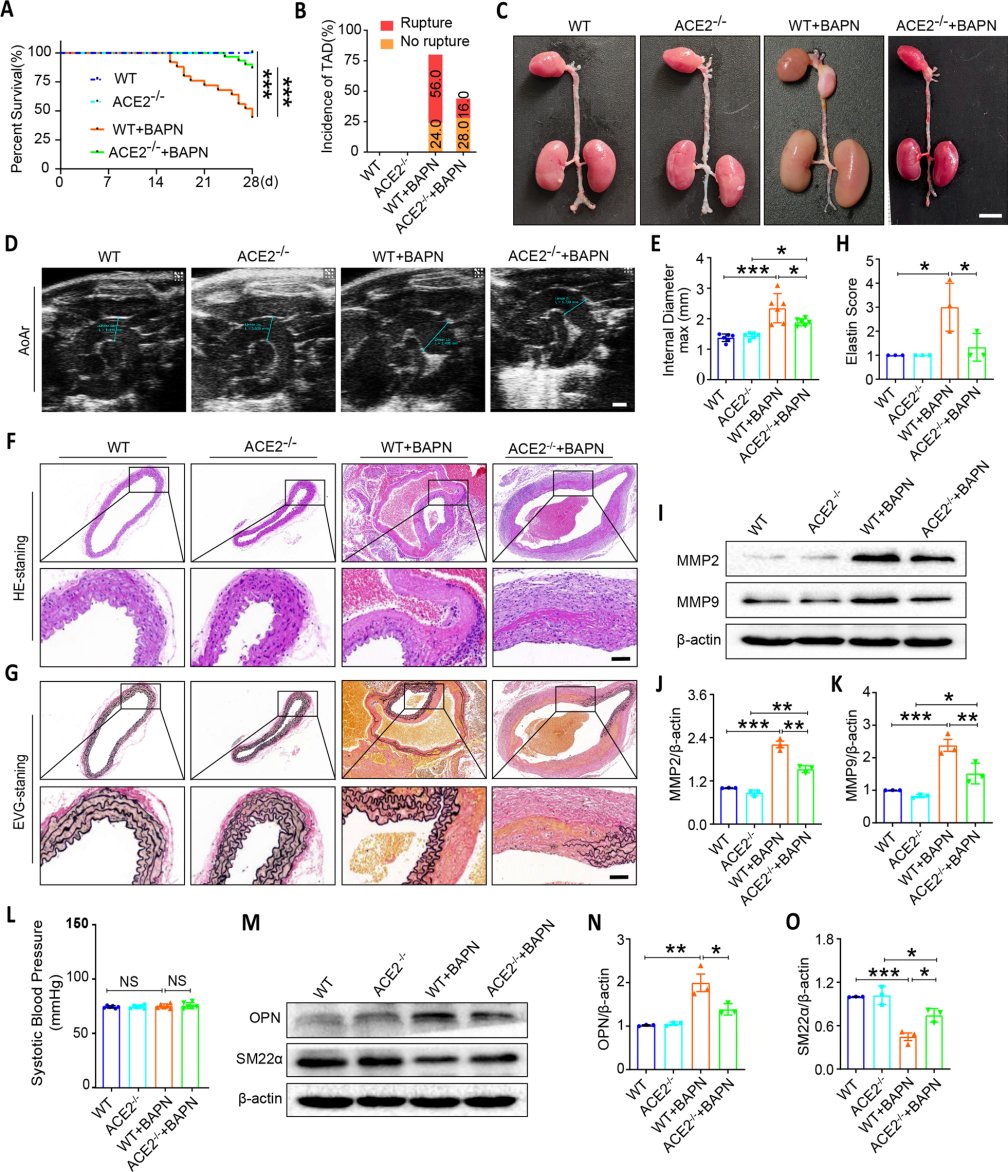
ACE2 deletion represses thoracic aortic dissection formation

To further verify the role of ACE2 in the pathological process of TAD, we generated ACE2 knockout mice and treated them with/without BAPN orally. Life-span analysis showed that BAPN administration provoked a remarkable morbidity and mortality in wild-type mice, whereas ACE2 deficiency significantly decreased the incidence and increased the survival rate (Fig. 3A, B). Macroscopic and thoracic aortic ultrasonography results on aortas demonstrated that BAPN promoted the formation of TAD and dilated aneurysms in the thoracic aorta in mice. Despite ACE2 deficiency itself did not affect the pathological process of TAD, it dramatically lowered BAPN-induced thoracic aortic dilation (Fig. 3C–E). Additionally, HE and EVG staining showed increased elastin score after BAPN administration, while ACE2 deficiency attenuated BAPN-induced pathology in the thoracic aorta (Fig. 3F–H). As shown in Fig. 3I–K, ACE2 deficiency inhibited BAPN-induced ECM degradation by up-regulating the protein levels of MMP2 and MMP9. However, regardless of whether the mice were orally administered with BAPN or knocked out of ACE2, there was no significant difference in their blood pressure (Fig. 3L). Concordantly, ACE2 deficiency promoted the expression of contraction-related gene SM22α, and inhibited synthesis-related gene OPN in BAPN-induced mice (Fig. 3M–O). Taken together, these results indicated that ACE2 deficiency inhibited BAPN-induced TAD formation.

### ACE2 activation exacerbates BAPN-induced pathology in thoracic aortic

**Fig. 4:**
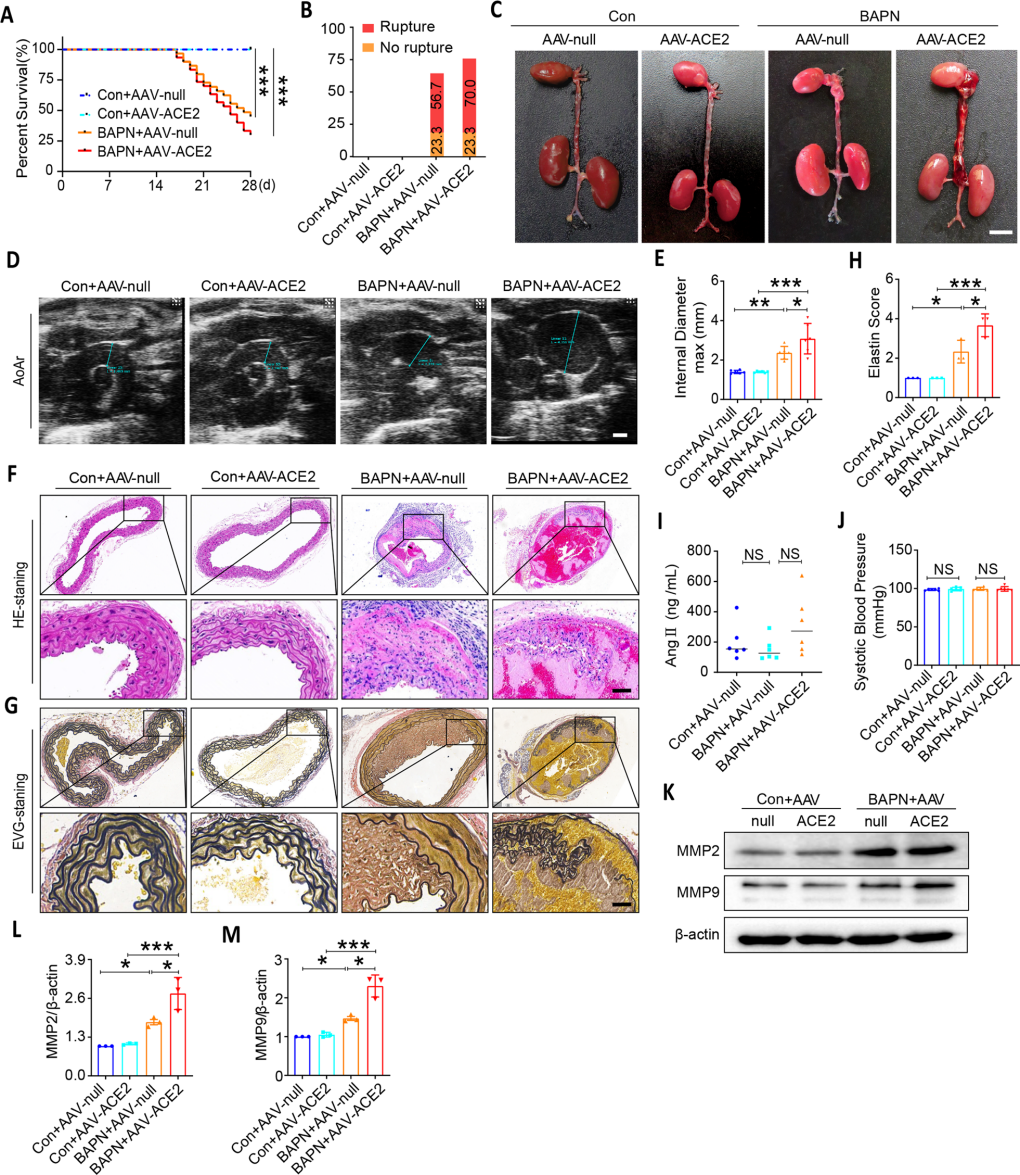
Overexpression of ACE2 promoted thoracic aortic dissection formation in mice

To further study the increased expression of ACE2 in BAPN-induced thoracic aortic tissue as in TAD patients, we treated mice with/without AAV-ACE2. As presented in Fig. 4A–E, ACE2 overexpression promoted the incidence and mortality of TAD, and increased maximal aortic diameters of thoracic aortic in BAPN-induced mice. In addition, HE and EVG staining showed increased elastin score in BAPN-induced thoracic aortic tissue, and ACE2 overexpression further exacerbated BAPN-induced pathology in thoracic aorta (Fig. 4F–H). While, as presented in Fig. 4I, the levels of AngII in peripheral blood plasma has no significant difference among the indicated groups. And the blood pressure has also no significant difference among the indicated groups (Fig. 4J). Immunoblotting in Fig. 4K–M showed that ACE2 activation promoted ECM degradation by increasing the expression of MMP2 and MMP9 proteins in BAPN-treated thoracic aortic tissues. However, ACE2 itself did not have any effect on MMP2 and MMP9 protein levels without BAPN. These results indicated that ACE2 aggravated BAPN-induced TAD.

### ACE2 overexpression aggravates inflammatory infiltration, promoted VSCMs phenotypic switch after BAPN administration

**Fig. 5:**
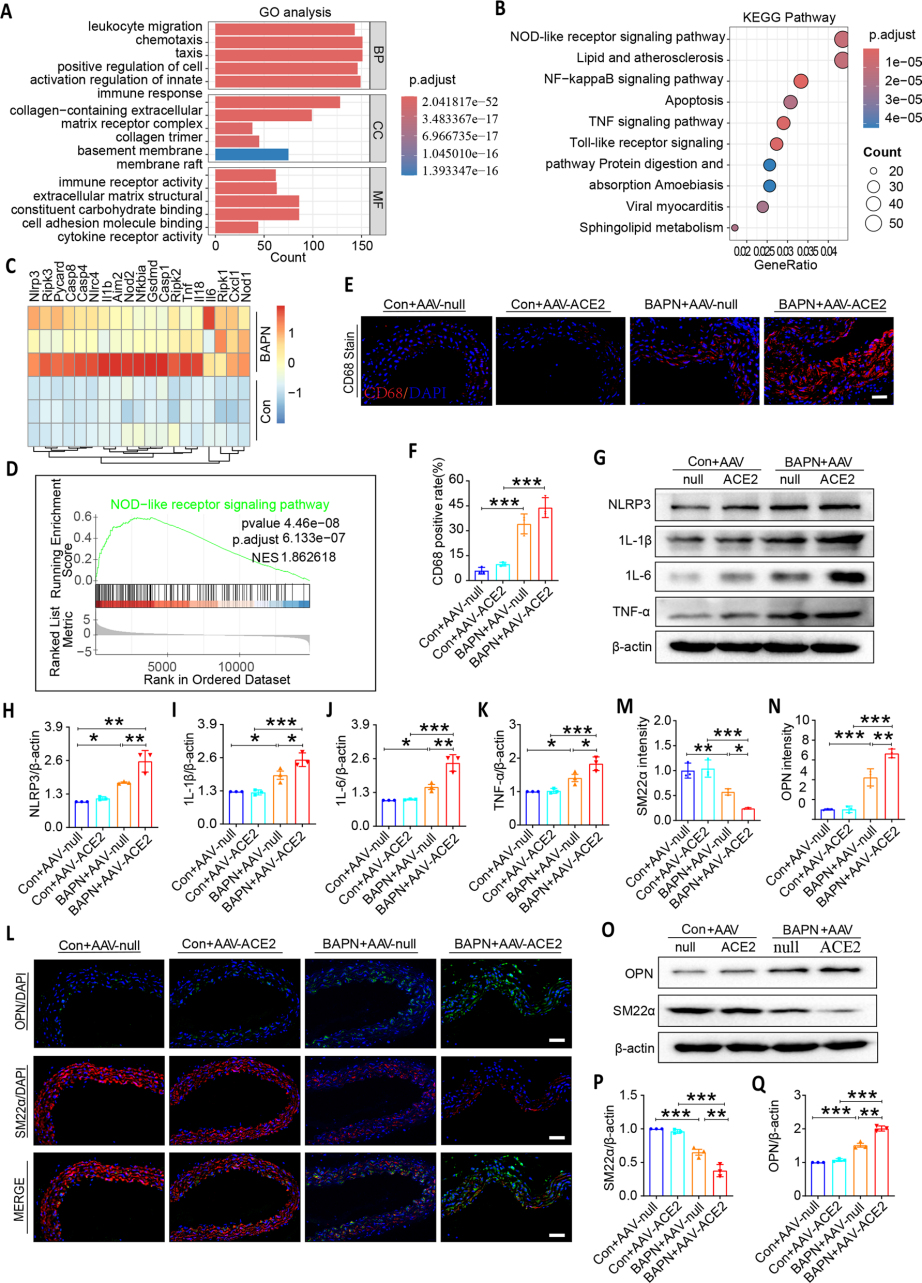
Overexpression of ACE2 promotes inflammatory reaction and exaggerates VSMC phenotypic switching in vivo

In the acute phase of aortic dissection, a large number of inflammatory cells infiltrate the vascular wall and secrete inflammatory factors, which can promote phenotype transformation of VSMCs. Meanwhile, when VSMCs switch from a contractile to a synthetic phenotype, synthetic VSMCs also secrete some inflammatory factors, which further exacerbate the inflammatory response. GO analysis showed that immune cells and ECM structural constituent were extensively enriched in the BAPN group compared with the con group (Fig. 5A). KEGG enrichment analysis indicated that the top differentially expressed genes were enriched in the inflammatory-related pathways, such as NLRPs, NF-kappaB signaling, and TNF signaling (Fig. 5B). Furthermore, the hierarchical clustering heatmap identified differentially expressed genes associated with the inflammatory response (*Nlrp3, Ripk3, Il1b, Aim2, Casp1, Tnf, Il18, Il6*) (Fig. 5C). And as shown in Fig. 5D, the NLRP signaling pathway was significantly upregulated in the BAPN group cluster.

To identify the molecular mechanisms by which ACE2 overexpression aggravates vascular remodeling, we examined vascular inflammation and the production of inflammatory cytokines in BAPN-induced thoracic aortic tissues. CD68 immunofluorescence staining, a macrophage biomarker, revealed that ACE2 further promoted BAPN-induced macrophage infiltration by increasing CD68-positive cells in mouse thoracic aortic tissues (Fig. 5E, F). Moreover, as shown in Fig. 5G–K, BAPN can increased the expression of pro-inflammatory cytokines of NLRP3, IL-1β, IL-6, and TNF-α in mouse thoracic aortic tissues compared with the control group. In particular, results in Fig. 5L–Q showed that the VSMC synthetic marker OPN was increased and the contractile marker SM22α was down-regulated in thoracic aortic tissues of mice suffering from TAD compared with the control group. Even though ACE2 itself did not have any effect on vascular remodeling of mouse arteries, it greatly promoted inflammation and VSCMs phenotypic switch in BAPN-induced mouse TAD model. These results suggested that ACE2 up-regulation increased the expression of pro-inflammatory cytokines and promoted VSCMs phenotypic switch in BAPN-induced mouse aortic tissues.

### SIRT3 was down-regulated in TAD patient and mice, and ACE2 activation lowered SIRT3 expression in BAPN-induced TAD mouse models

**Fig. 6:**
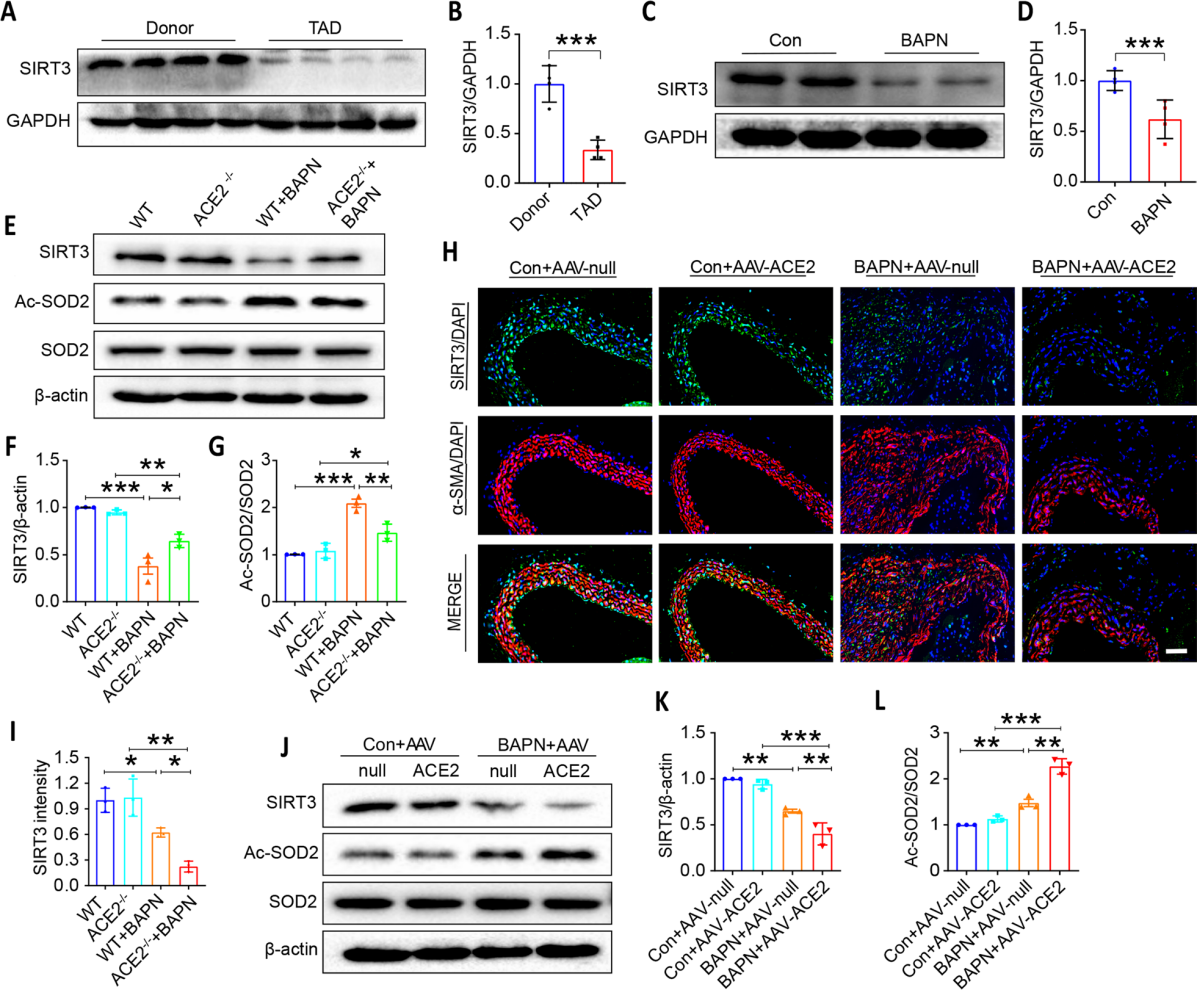
SIRT3 expression is decreased in TAD, and SIRT3 was regulated by ACE2 in the process of TAD

Previous reports have shown that SIRT3 overexpression attenuated aneurysm formation and decreased aortic expansion induced by Ang II (Qiu et al. 2021). In our study, western blotting analysis in Fig. 6A, B revealed that SIRT3 expression was significantly inhibited in aortic vasculature of TAD patient compared with the Donor group, and the result was consistent in mouse aortic vasculature (Fig. 6C, D). Moreover, ACE2 deficiency reversed the down-regulation of SIRT3 expression and the up-regulation of Ac-SOD2 expression induced by BAPN in TAD mouse model (Fig. 6E–G). Furthermore, immunofluorescent staining showed that ACE2 activation intensifies the decrease of SIRT3 expression in BAPN-induced mouse aortic tissue (Fig. 6H, I). Similar results were obtained from western blotting analysis data (Fig. 6J, K). Meanwhile, we found that Ac-SOD2 was significantly increased in BAPN-treated mice compared with the con + AAV-null group. ACE2 activation further elevated Ac-SOD2 expression (Fig. 6J and L). These data imply that ACE2 activation lowered SIRT3 expression and aggravated BAPN-induced aortic injury via elevated Ac-SOD2 expression.

### SIRT3 inhibition aggravates thoracic aortic vasculature lesion in BAPN-induced TAD mouse models

**Fig. 7:**
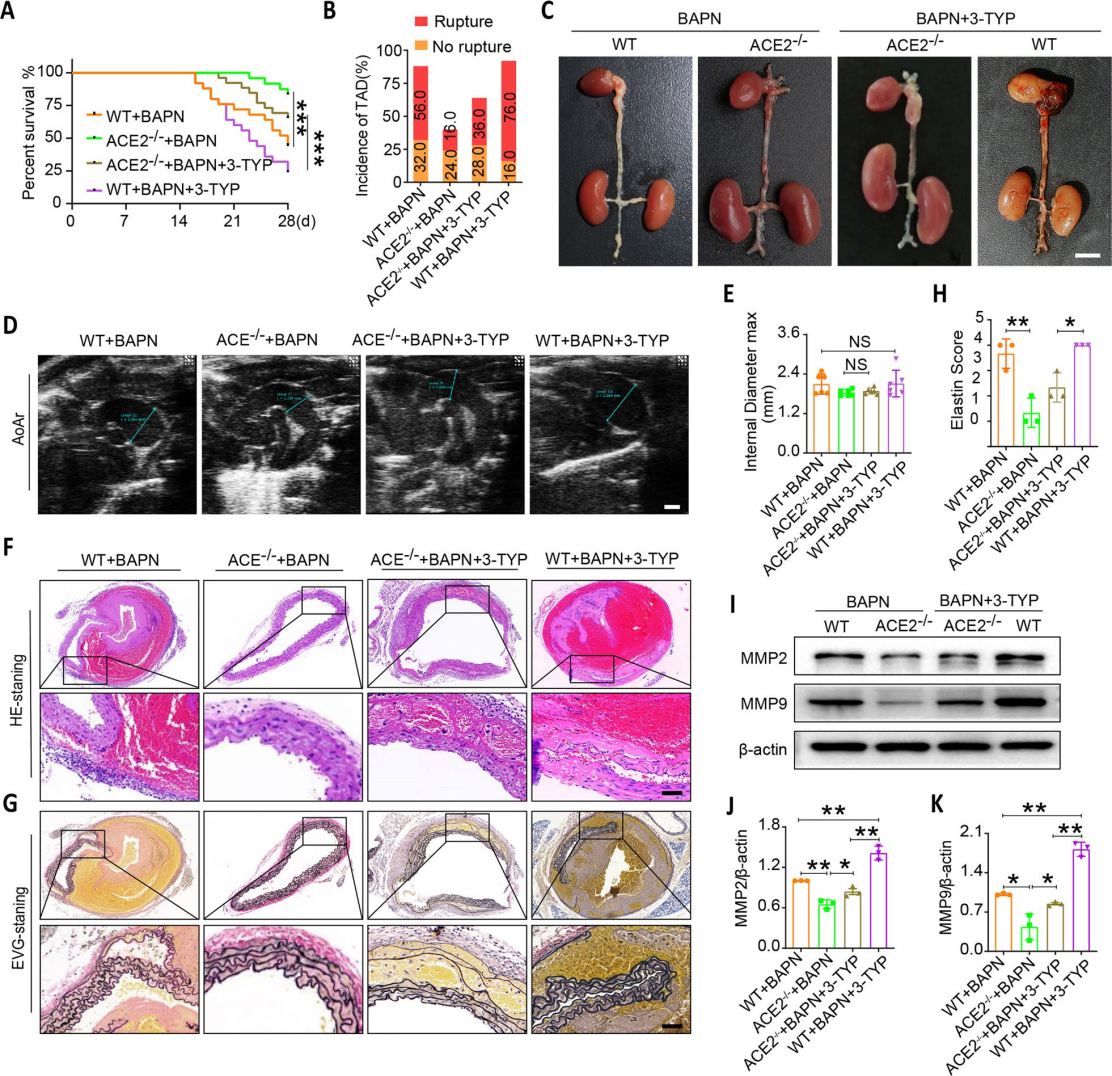
Inhibiting SIRT3 promotes thoracic aortic dissection formation in ACE2-/- mice

As shown in Fig. 7A–E, ACE2 knockout significantly increased survival and decreased the incidence of TAD in BAPN-treated mice. However, inhibition of SIRT3 exacerbated BAPN-induced thoracic aortic injury by increasing mortality, the incidence of TAD and expansion of the maximal aortic diameters in the BAPN-induced mouse model with ACE2 deficiency. In addition, H&E and EVG staining results revealed that ACE2 deletion markedly improved the histological morphology and protected the lumen elastin fibers of the thoracic aorta in the mice after BAPN treatment. Of interest, SIRT3 inhibition reversed the protective effect of ACE2 deletion in the BAPN-treated mouse TAD model (Fig. 7F–H). The decreased expression of MMP2 and MMP9 in the aortic walls from ACE2 deletion in BAPN-induced TAD mice was further confirmed by western blotting. However, SIRT3 inhibitor treatment significantly increased the expression of MMP2 and MMP9, thus increasing the degradation of the ECM (Fig. 7I–K). These findings suggested that SIRT3 participated in the protective role of ACE2 deletion in the BAPN-induced TAD mouse model.

### SIRT3 inhibition partially offsets the protective role of ACE2 deletion on TAD mouse models by elevating pro-inflammatory cytokines expression

**Fig. 8:**
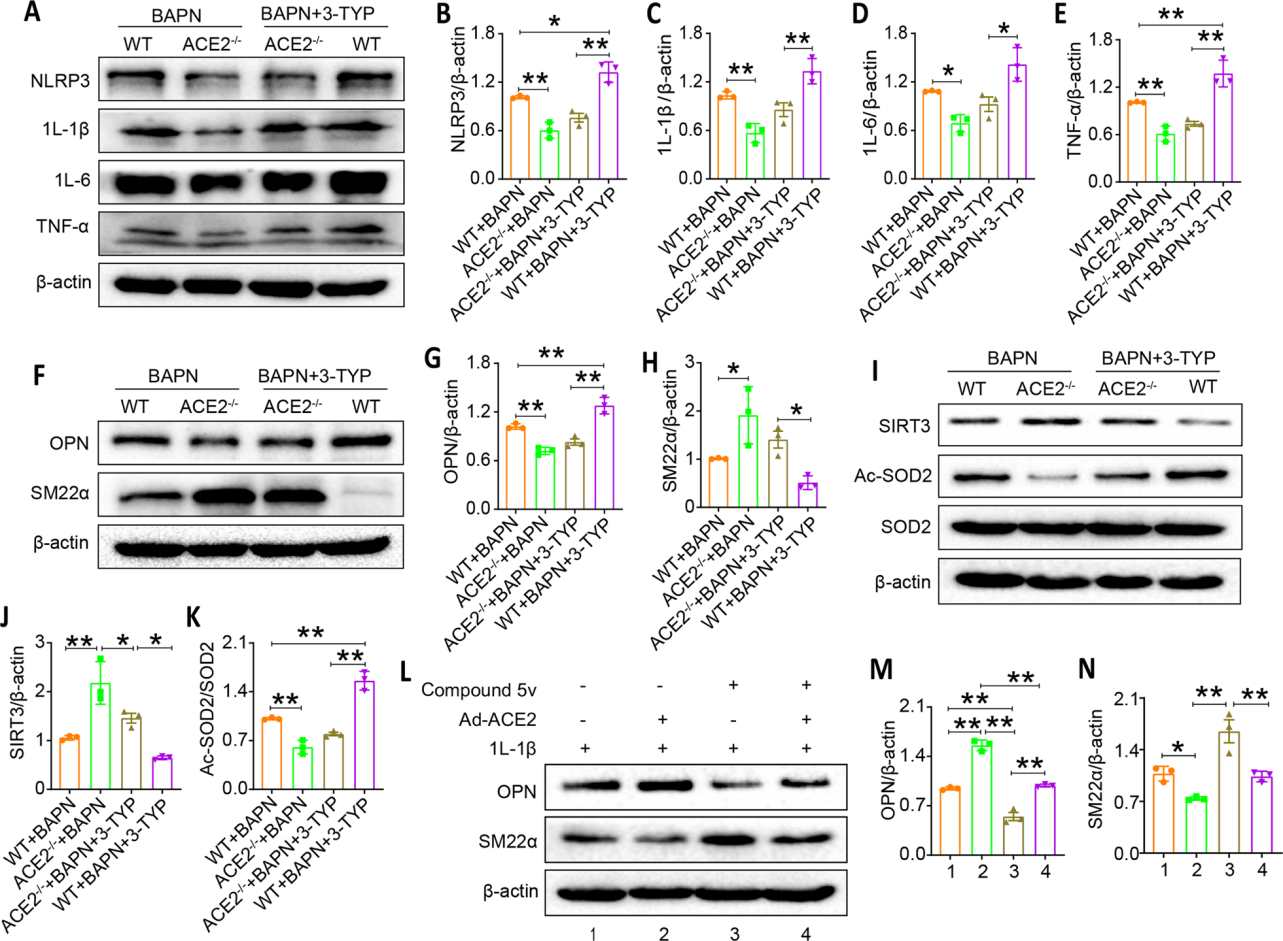
Inhibiting SIRT3 promotes inflammation and VSMC phenotypic switching in aortic sections in ACE2-/- mice, and activating SIRT3 InhibitsVSMC phenotypic switching in 1L-1β treated VSMC transfected with Ad-ACE2

To determine whether inhibition of SIRT3 underlies ACE2 deficiency against BAPN-induced TAD injury, the effects of SIRT3 down-regulation with 3-TYP on TAD were examined in wild type and ACE2^−/−^ mice. Data in Fig. 8A–E demonstrated that ACE2 deletion reduced the expression of pro-inflammatory cytokines of IL-1β, IL-6 and TNF-α and NLRP3 in mouse thoracic aortic tissues compared with the WT + BAPN group. SIRT3 inhibition further elevated inflammasome NLRP3 expression, which exacerbated BAPN-induced ACE2^−/−^ mice TAD lesion. While SIRT3 inhibition reversed the protective role of ACE2 deletion on the BAPN-induced TAD mouse model by provoking the secretion of pro-inflammatory cytokines. Similar results were observed in Fig. 8F–H, treated the ACE2^−/−^ mice with BAPN, VSMC synthetic marker OPN was down-regulated and contractile marker SM22α was increased in thoracic aortic tissues of TAD mice, but SIRT3 inhibition reversed the results. Moreover, ACE2 deficiency significantly increased SIRT3 expression and reduced Ac-SOD2 expression induced by BAPN in TAD mouse model, while SIRT3 inhibition inhibited the expression of SIRT3 and promoted expression of Ac-SOD2 in BAPN induced ACE2^−/−^mice (Fig. 8I–K). These data indicated that SIRT3 was likely to be responsible for ACE2^−/−^ -mediated aorta vascular protective effect, inhibition of phenotype transformation of VSMCs and inflammation in TAD modle underneath BAPN challenge.

To further confirm that SIRT3 overexpression can reverse the effects of ACE2 overexpression. We performed in vitro experiments. The results showed that ACE2 overexpression exacerbated VSMC switch to a synthetic phenotype mediated by IL-1β. While, SIRT3 agonist significantly downregulated the expression of OPN, upregulated the expression of SM22α in IL-1β stimulated VSMC. However, when ACE2 is overexpressed and SIRT3 is activated simultaneously, activating SIRT3 significantly improved the loss of contractile proteins in VSMC induced by overexpression of ACE2 (Fig. 8L–N). These data indicated that SIRT3 can offset the negative effects of ACE2 overexpression on VSMC transition from contraction to synthesis.

## Discussion

**Fig. 9:**
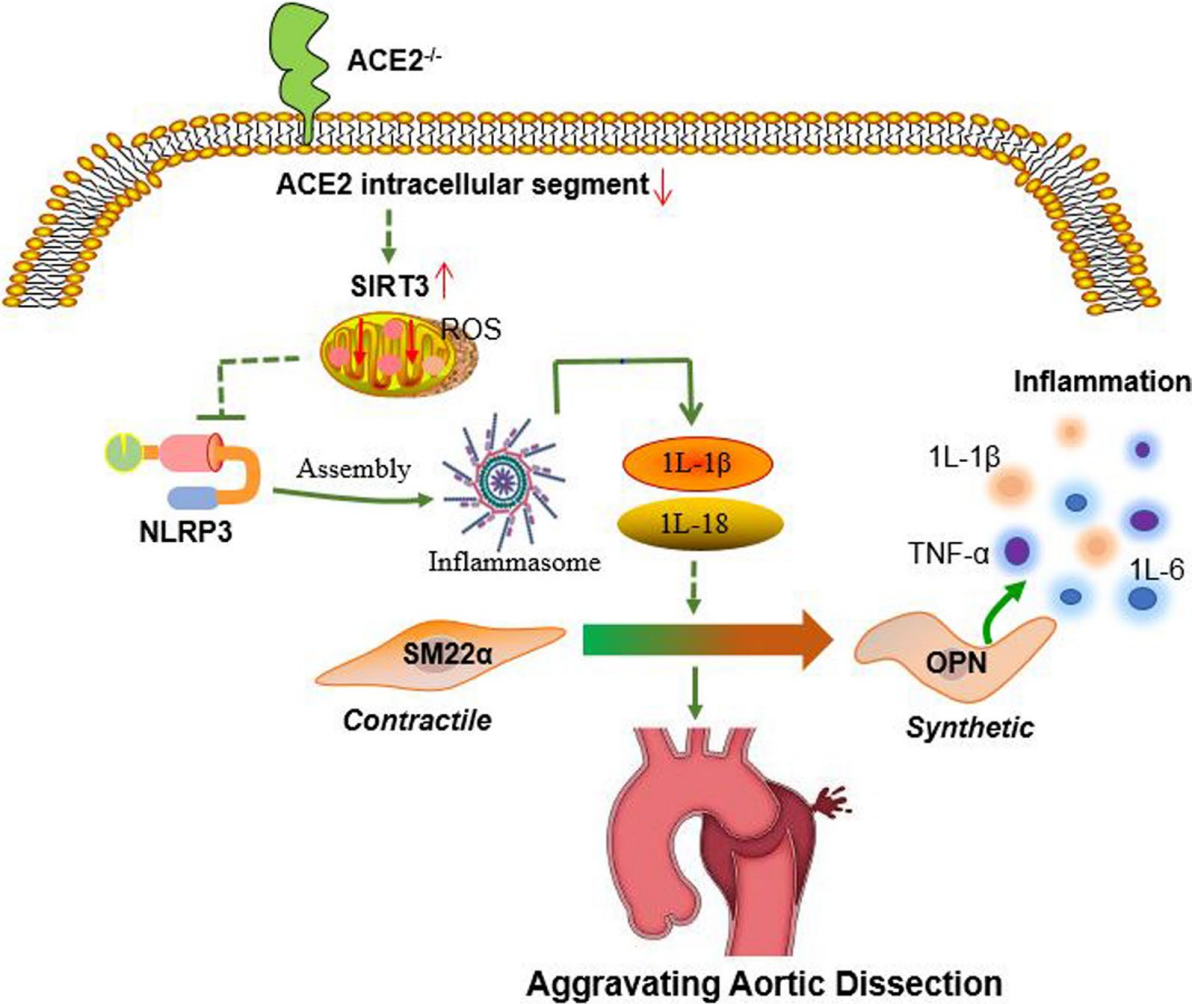
Summary figure. The mechanistic showing that ACE2 deficiency inhibits TAD by enhancing SIRT3 mediated inhibition of inflammation and VSCMs phenotypic switching

Despite numerous studies have focusing on the explanation of mechanisms of TAD, which remains a major cause of mortality and morbidity, surgical treatment remains the optimal option for TAD treatment (Rylski et al. 2023; Hameed et al. 2023). Moreover, surgical procedures often come with various complications such as the distal re-entry tears of AD, stroke, and even death (Cao et al. 2023a, b; Zhang et al. 2023a, b). Although the molecular mechanism has been investigated, till, no effective therapeutic drug has been found to fully reverse or inhibit TAD. The salient findings in this study revealed that ACE2 participated in the protective role of SIRT3 against inflammation and VSMC phenotypic switch in TAD patients and BAPN-induced rodents. This finding offers a potential drug target for an effective pharmacological treatment for TAD (Fig. 9).

The expression of ACE2 is abundant in various organs including the heart and vascular systems, and it plays a pathological and physiological role in cardiovascular systems (Pan et al. 2018; Bian and Li 2021). Most previous studies have confirmed that ACE2 primarily exerts cardiovascular protection by lowering blood pressure (Pan et al. 2018). However, the function of ACE2 in the BAPN-induced TAD remains elusive. Increasing evidence suggests that lowering blood pressure dose not ameliorate aortic rupture and dilatation in BAPN-administered mice (Sawada et al. 2022; Kurihara et al. 2012). Here, our data revealed that ACE2 in aorta was increased both in human TAD samples and in mouse TAD models. ACE2 up-regulation markedly aggravated BAPN-induced morbidity, mortality, and thoracic aortic dilation. Much to our surprise, ACE2 up-regulation did not lead to a decrease in serum AngII and blood pressure. Whereas, ACE2 deficiency significantly decreased the incidence, and attenuated BAPN-induced thoracic aortic dilation, attenuated BAPN-induced pathology in the thoracic aorta. Collectively, these results indicate that ACE2 participated in the pathology of BAPN-induced TAD.

As is known to all that VSMC homeostasis plays a crucial role in vascular remodeling and is associated with several common vascular disorders including hypertension, atherosclerosis, and TAD.

VSMCs exhibiting a loss of contractile proteins, loss and accumulation of inflammation were important indicators of TAD (Xia et al. 2020; Pan et al. 2022). Our transcriptomic results confirm this. Accordingly, this was verified by immunoblotting in patients with TAD and BAPN-induced mice. Our further results denoted that ACE2 overexpression further promoted extracellular matrix degradation, inflammatory injury and aberrant VSMC switch to a synthetic phenotype. While, genetic ablation of ACE2 significantly reversed those results. Our studies add new knowledge to the field of VSMC phenotypic switch in TAD pathology.

VSMC phenotypic switching is a complex pathological process, despite significant progress in the past few decades, the underlying mechanisms remain incompletely elucidated. Interestingly, in this study, with the increase of ACE2 levels, the expression of SIRT3 proteins showed an opposite trend in BAPN-induced mice. We propose that SIRT3 is a downstream regulator of ACE2 for vascular inflammation and VSMC phenotypic switch associated with TAD. The further data in this study demonstrated that overexpression ACE2 suppressed the protein level of SIRT3 in TAD mice aortic tissues, while ACE2 deficiency alleviated the inhibition of BAPN on SIRT3 in aortic specimens. Moreover, inhibition of SIRT3 offset the protective role of ACE2 deficiency against BAPN-induced pathology in the thoracic aortic. Therefore, it is important to understand the molecules and mechanisms that mediate the activation or inhibition of ACE2 by SIRT3 in BAPN-induced mice.

SIRT3 are a family of proteins with enzymatic activity, is involved in maintaining mitochondrial integrity, homeostasis, and function and plays an important protective role in cardiovascular health, including myocardial ischemia–reperfusion, cardiac hypertrophy, and even heart failure (Zhang et al. 2023a, b; Wang et al. 2023; Deng et al. 2021). In addition, it was reported that SIRT3 upregulation attenuates AngII-induced hypertrophy by inhibiting vascular oxidative stress and endothelial dysfunction. However, deletion of SIRT3 suffered from vascular inflammation and increased vascular permeability in AngII-induced vascular injury (Dikalova et al. 2020; Qiu et al. 2021; Li et al. 2019). The mechanism of SIRT3 involved in these vascular protections is related to the mitochondrial homeostasis. Moreover, SITR3 can be directly or indirectly activated by some molecules. For instance, some studies have reported that Apelin can exert cardiovascular protection by activating SIRT3 to regulate mitochondrial homeostasis (Wang et al. 2024; Ni et al. 2020). Especially, Apelin induces a SMC phenotypic transition towards the synthetic phenotype in atherosclerosis (Cardoso Dos Santos, et al. 2023). In our study, ACE2 deficiency alleviated the inhibition of BAPN on SIRT3 in aortic specimens. Whether Apelin may be an intermediate link involved in this pathological process still needs further verification. Besides, SIRT3 depletion can induce mitochondrial damage due to SOD2 acetylation and SOD2 inactivation in vascular dysfunction and hypertension (Dikalova et al. 2020). As showed in our results, when SIRT3 was inhibited, the acetylation level of SOD2 significantly increased. While, the deacetylation site of lysine residues in SIRT3 has not been elucidated, which is a limitation of our research.

Taken together, we demonstrated the promising therapeutic effects that ACE2 deficiency mediated the protective effect of SIRT3 against VSMCs phenotypic switch and vascular inflammation in BAPN-induced TAD. These results improve our understanding of the relationship of the RAS system and sirtuin family. These results provide potential therapeutic strategies to address vascular diseases for TAD.

## Conclusion

1. VSMCs undergo phenotypic modulation and inflammation promotes phenotype transformation of VSMCs in the pathological development process of TAD, meanwhile stimulating the expression of ACE2.
2. ACE2 deficiency attenuates the development of TAD induced by BAPN, and ACE2 activation exacerbates BAPN-induced pathology in thoracic aortic
3. ACE2 deficiency inhibits inflammatory infiltration and VSCMs phenotypic switch through activation of SIRT3 signal pathway after BAPN administration.

## Supplementary Information

Supplementary Material 1.

## Data Availability

All data used in this study are available from the authors on reasonable request.
