# Supplementary material for: ACE2 deficiency inhibits thoracic aortic dissection by enhancing SIRT3 mediated inhibition of inflammation and VSCMs phenotypic switch
Source: Mol Med. 2024 Sep 19;30:154. doi: 10.1186/s10020-024-00926-4 (PMC11414040; doi:10.1186/s10020-024-00926-4)
Supplement: Supplementary file 1 — Supplementary Material 1. [file 10020_2024_926_MOESM1_ESM.pdf]

The knockout mice were named as C57BL/6J-Ace2^{em1cyagen}, and the serial number is KOCMP-70008-Ace2. The generations of knockout mice were confirmed by polymerase chain reaction (PCR) using genotyping primers presented in follow table. And the PCR results were showed in follow figure.

Supplemental table

Table S1 Genotyping primers for PCR

Genotyping primers for PCR
**PCR Primers 1 (Annealing Temperature 60.0 °C):**
Forward primer (F1): 5'-GACGTTGTGCATTGACTGTTCTA-3'
Reverse primer (R1): 5'-CTACATTACCAGGCAAATGGAAGT-3'
Targeted allele: 569 bpPCR
**Primers2 (Annealing Temperature 60.0° C):**
F1: 5'-GACGTTGTGCATTGACTGTTCTA-3'
R2: 5'-TACTTTGCTGAGGGTCAAGGTTTA-3'
Product size: 560 bp
Homozygotes: one band with 569 bp
Heterozygotes: two bands with 569 bp and 560 bp
Wildtype allele: one band with 560 bp

Supplemental Figures

Supplemental Figure 1

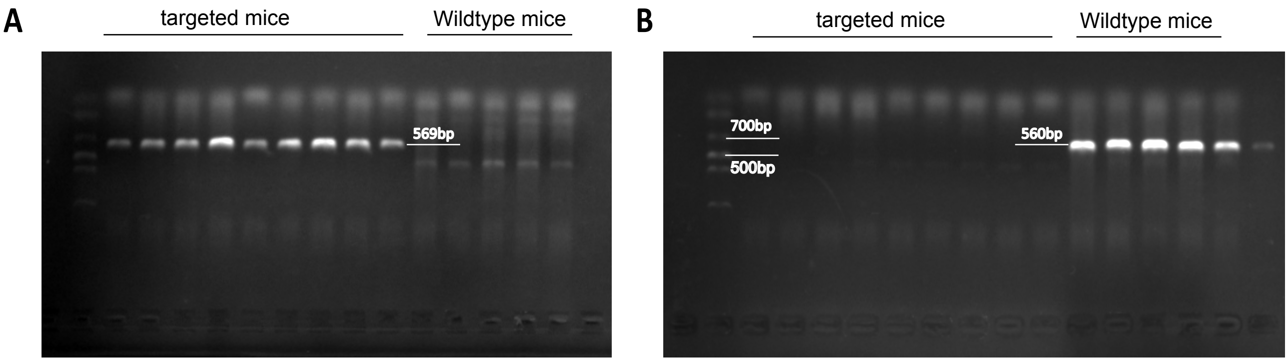

Supplemental Figure 2

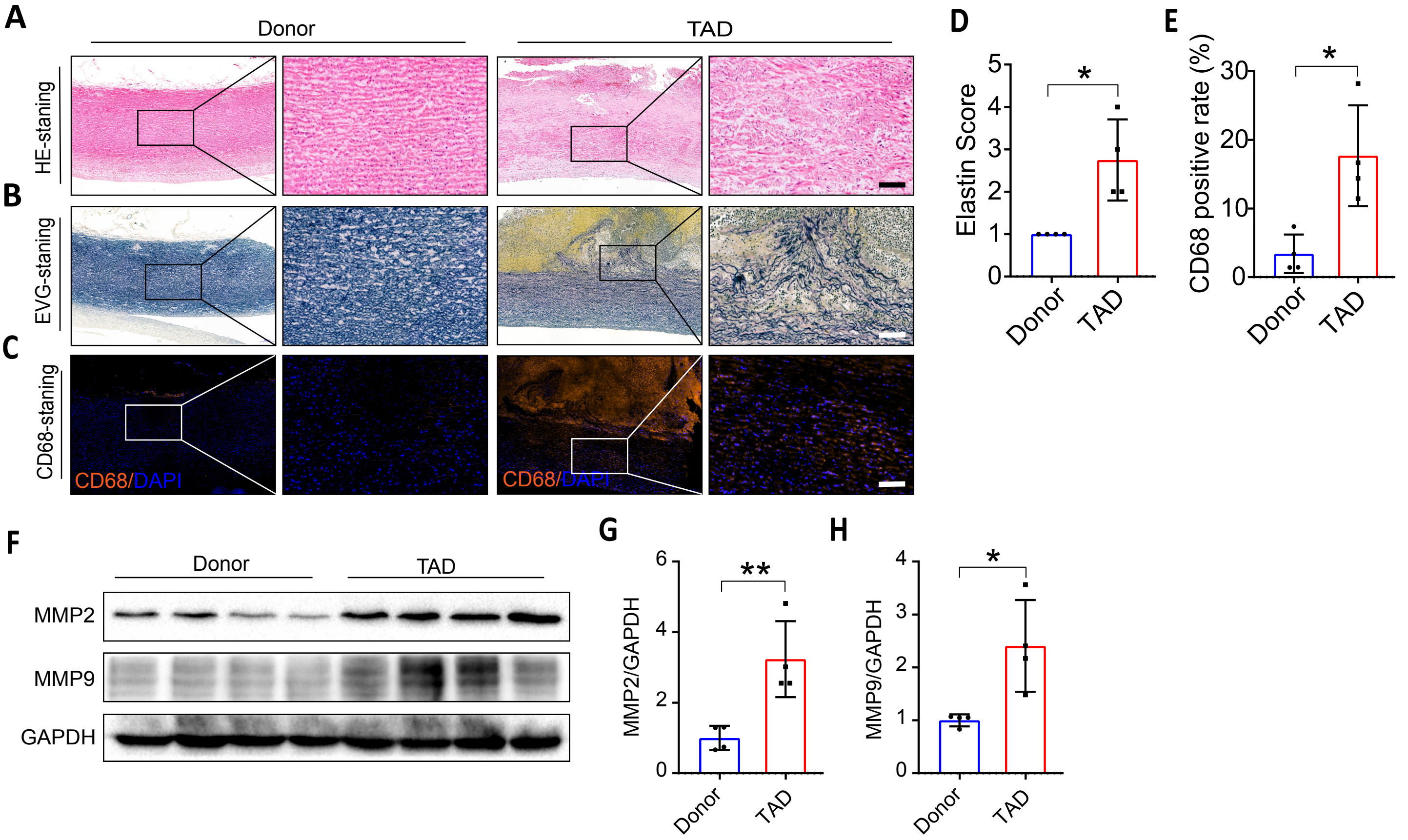

Supplemental Figure Legends

Figure S1. Results of PCR validation in ACE2 KO and Wildtype mice.
A. Representative PCR genotyping results of two groups mice using PCR Primers1. **B.**Representative PCR genotyping results of two groups mice using PCR Primers2.

Figure S2. Staining of main pathological changes in aortic sections from clinical and experimental specimens of TAD and Donor.
A. Representative images of histological staining with hematoxylin in aortic sections from TAD and Donor (bars=100 μm). **B.** Representative images of histological staining with elastica-van Gieson (bars=100 μm). **C.** Representative images of CD68 immunofluorescence staining (bars=100 μm). **D.** Elastin score (n = 4 per group). **E.** Number of CD68-positive cells per square millimeter in each group (n = 4 per group). **F.** Representative Western blots of MMP2 and MMP9. **G and H.** Quantified expression of MMP2 and MMP9 (n = 4 per group). The results are presented as the mean ± SD; $^{*}P < 0.05$, $^{**}P < 0.01$.
